# Research on Storm-Tide Disaster Losses in China Using a New Grey Relational Analysis Model with the Dispersion of Panel Data

**DOI:** 10.3390/ijerph14111330

**Published:** 2017-11-01

**Authors:** Kedong Yin, Ya Zhang, Xuemei Li

**Affiliations:** 1 School of Economics, Ocean University of China, Qingdao 266100, China; yinkedong@ouc.edu.cn (K.Y.); zhangya1992@126.com (Y.Z.); 2 Ocean Development Research Institute, Major Research Base of Humanities and Social Sciences of Ministry of Education, Ocean University of China, Qingdao 266100, China

**Keywords:** dispersion, grey relational analysis, sustainable development on marine economics, storm-tide disaster

## Abstract

Owing to the difference of the sequences’ orders and the surface structure in the current panel grey relational models, research results will not be unique. In addition, individual measurement of indicators and objects and the subjectivity of combined weight would significantly weaken the effective information of panel data and reduce the reliability and accuracy of research results. Therefore, we propose the concept and calculation method of dispersion of panel data, establish the grey relational model based on dispersion of panel data (DPGRA), and prove that DPGRA exhibits the effective properties of uniqueness, symmetry, and normality. To demonstrate its applicability, the proposed DPGRA model is used to research on storm-tide disaster losses in China’s coastal areas. Comparing research results of three models, which are DPGRA, Euclidean distance grey relational model, and grey grid relational model, it was shown that DPGRA is more effective, feasible, and stable. It is indicated that DPGRA can entirely utilize the effective information of panel data; what’s more, it can not only handle the non-uniqueness of the grey relational model’s results but also improve the reliability and accuracy of research results. The research results are of great significance for coastal areas to focus on monitoring storm–tide disasters hazards, strengthen the protection measures of natural disasters, and improve the ability of disaster prevention and reduction.

## 1. Introduction

### 1.1. Grey Relational Analysis

Grey relational analysis (GRA) is an important branch of grey system theory (GST) that is used to determine the relational degree among factors according to similarities in their geometry. Higher similarities in the geometric shape of a line or curve suggest higher relational degrees among the study factors [1]. As GRA theory has matured, it has been widely used as a research tool in societal economic and production practices. According to different research perspectives, the relational GRA model can be categorized as (1) proximate, (2) similar and (3) comprehensive.

Proximate relational models primarily measure the relational degree among factors according to the distance between sequences. Examples of proximate relational models include Deng’s general relational model and the absolute relational model [1]. In Deng’s general relational model, proximity magnitudes and trends among factors were measured using Euclidean distances or relational coefficients that defined the general relational degree [1]. Numerous researchers have subsequently modified and advanced Deng’s original methods for determining relational degree [1,2,3,4,5,6,7,8]. As a subset of proximate relational models, absolute relational models generally use the area difference or pattern distance to measure proximity. Various forms of the absolute relational degree include the first absolute relational degree [9], the second absolute relational degree [10], the generalized absolute relational degree [11] and the relevant improving model [12,13]. Researchers have subsequently developed new relational models [14,15,16,17,18,19,20].

Unlike proximate relational models that measure relational degree according to the distance between sequences, similar relational models measure relational degree according to the sequence trend using increment, slope, velocity and acceleration variables to describe geometric characteristics and trends. A relational coefficient model was constructed using Euclidean and fuzzy distances of a signature series. Various forms of similar relational degree include the B-correlation degree [21], the C-correlation degree [22], the T-correlation degree [23], the slope relational degree [24] and its corresponding improving model [25,26,27,28,29,30,31,32,33].

Combining aspects of proximate and similar relational models, comprehensive relational models measure the proximity degree of the distance and the similarity degree of the trend. Shi combined a slope relational degree with a general relational degree according to the linear weight of two models to develop a point and oblique relational degree [34]. Chen et al. developed a trend relational matrix by comparing subset and reference subset distances that reflected not only the magnitude of the factor change rate but also the proximity degree between factors [35]. Based on a grey absolute relational model, Liu et al. developed a GRA model using two different perspectives of similarity and proximity [36]. Jiang et al. used the polygon area of two curves to measure their distance proximity and geometric similarity and subsequently developed a GRA model [37].

Over time, GRA theory has gradually expanded to three-dimensional space. Zhang et al. used panel data in three-dimensional space to develop a multidimensional relational degree, which extended the matrix-based grey absolute relational degree [38]. Wu et al. proposed a grey convex relational degree for three-dimensional panel data based on the grey convex relational degree for two-dimensional data and the Hessian matrix approximation for a disperse sequence [39]. Qian et al. developed a grey matrix relational model to measure the cross-sectional similarity of indexes based on panel data [40]. Liu et al. described the geometrical features of panel data in three-dimensional space using a grid method and developed grid relational coefficients; according to arithmetic averages, a grey grid relational model was subsequently established [41]. Li et al. synthesized three different types (deviation, difference and separation) to establish an index relational analysis model [42]. Cui et al. expanded traditional vector spaces to matrix spaces to develop a grey matrix similarity relational model based on panel data [43]. Most recently, Wu et al. developed a similar and proximate relational model based on the angles and distances of panel data space vector, respectively [44].

To summarize, existing GRA models have been developed based on geometric features, slope, area, distance, angle et al., and gradually extended to three-dimensional space, which is more authentic and comprehensive for reflecting the relational degree of research objects. At present, GRA models have been widely applied in numerous fields related to economy [45,46,47], management [48,49], disaster risk [50], society [51,52], industry [53,54], physics [55,56], chemistry [57,58], transportation [59], ecology [60], geology [61] and aeronautics and astronautics [62].

### 1.2. Storm-Tide Disaster Losses

Storm surge is an abnormal rising phenomenon of sea level that is caused by strong atmospheric disturbances during tropical and extratropical cyclones (typhoons or hurricanes) [63]. Storm surge disaster loss refers to natural disasters, social disasters, economic disasters and environmental disasters and other losses collectively caused by storm surge movement and the evolution. China experiences frequent storm surges throughout the year and from southern to northern coastlines, which leads to incalculable losses of human life and property.

Most prior studies related to storm-tide disaster losses focused on disaster hierarchies, direct economic loss assessments and economic loss assessments associated with specific ecological or other environments. Ye et al. established a discriminant function for typhoon storm-tide disaster hierarchies in Guangdong by applying pattern recognition to storm-tide disaster hierarchies [64]. Jain et al. established a new model that synthesized four models of typhoon risk and changes in buildings, building vulnerability and economic income [65]. Liang analyzed Hainan Island coastal areas that were vulnerable to storm-tide disasters and summarized the relationships among various hazard-affected bodies and the elevation and submergence depths of carriers [66]. LisaR. Kleinosky et al. used SLOSH and DEM model to analyze the exposure of different intensity hurricane storm surges and floods at different sea level levels in the Hampton Roads region of Virginia Ji et al. proposed a fuzzy comprehensive evaluation method for storm-tide disaster loss hierarchies based on the concept of fuzzy disaster degree [67].

Based on the economic connotation of storm surge relief, Zhao et al. analyzed the economic impact of storm surge relief using supply and demand curves and concluded that greater storm surge rescue efforts resulted in lesser farming losses [68]. Zhao et al. used coupling analysis to describe the interaction mechanism between storm-tide disaster losses and regional economic growth systems [69]. In a series of studies, Yin et al. established an index system for storm-tide disaster economic losses according to their composition and concurrently evaluated storm-tide disaster losses using principal component analysis (PCA) [70,71,72]. Subsequently, they determined index weights using an analytical hierarchy process (AHP) and entropy value method and established a classification evaluation model for storm-tide disaster societal economic losses.

With a focus on specific ecological environments, McInnes et al. used Monte Carlo simulation methods to quantify the risk of storm-tide disasters in the Fiji Islands under global climate change [73]. Zhao et al. introduced the grey relational method of risk evaluation in meteorological disaster losses based on historical disaster data in China and apply the improved grey relational analysis model to the risk evaluation of rainstorm and flood disaster losses [74]. Lapidez et al. calculated the maximum probable storm surge height for every coastal locality by running simulations of Haiyan-type conditions [75]. Zhao et al. evaluated fishery losses based on prior research regarding storm-tide disasters [76]. Saha explored the dynamics of disaster-induced risk resulted from tropical Cyclone Aila and the influences of various socioeconomic, environmental, institutional and geographical factors on escalating disaster risk [77]. Most recently, Takagi et al. examined the impact of floodwaters on Leyte Island in the Philippines caused by the Typhoon Haiyan storm surge in 2013 [78]. Based on relevant research and experience, Kang et al. established a loss evaluation model of farmland yield caused by sea level rise and storm surges [79]. Zhang et al. assessed the vulnerability of Yuhuan County based on land use, then evaluated the overall storm surge risk for Yuhuan County [80].

To summarize, prior research has focused on the characteristics, cause and hierarchy of storm surge events. Some studies assessed post-disaster economic losses. However, these assessments considered aggregate economic losses; the different types of losses and the relationships among them were not considered.

### 1.3. Research Motivation and Scope

The study on the correlation of storm disaster losses contributes to the cognition of relationship between storm surge losses and will provides a scientific basis for minimizing these losses, in turn, minimzse associated economic losses and promote development of marine economy. Limited information currently exists that relates different types of storm-tide disaster losses to direct economic losses. Both the occurrence of storm surge and its associated losses are random. As such, supporting data to establish these relationships may not be continuous (data may be missing for select years or areas), which may lead to limited sample sizes and compromised data quality.

In response to these limitations, GRA models do not require large or regular data samples and hence have an important practical significance when investigating China’s storm-tide disaster losses. Conventional GRA models, however, have some shortcomings. For different connections or object orders in panel data, a conventional GRA model may produce non-unique results. Instead, we proposed a new GRA model based on the dispersion of panel data (DPGRA). Specifically, the DPGRA model was developed based on the dispersion of panel data. The proposed DPGRA model was used to relate direct economic losses with mariculture, coastal engineering, death toll, ship and collapsed home losses as well as storm surge frequency in China’s coastal areas in five main provinces. Correlations among these five provinces were also considered based on these seven indicators. This study verified through application that the proposed DPGRA model was valid and feasible and provided a theoretical basis for the prevention and control of future storm-tide disaster losses.

Following this introductory information, this paper describes the theoretical basis for panel data correlation analysis and demonstrates the potential for non-unique results from conventional GRA models based on panel data (Section 2). Next, the paper describes the proposed DPGRA model including its properties and capabilities for overcoming conventional GRA model shortcomings (Section 3). Section 4 presents verification results that demonstrate the proposed DPGRA model’s validity and feasibility using panel data from storm-tide disaster events in China’s coastal areas (i.e., empirical analysis results). The paper concludes by summarizing significant findings and describing topic areas for future research (Section 5).

## 2. Theoretical Basis for Panel Data Correlation Analysis

**Figure 1:**
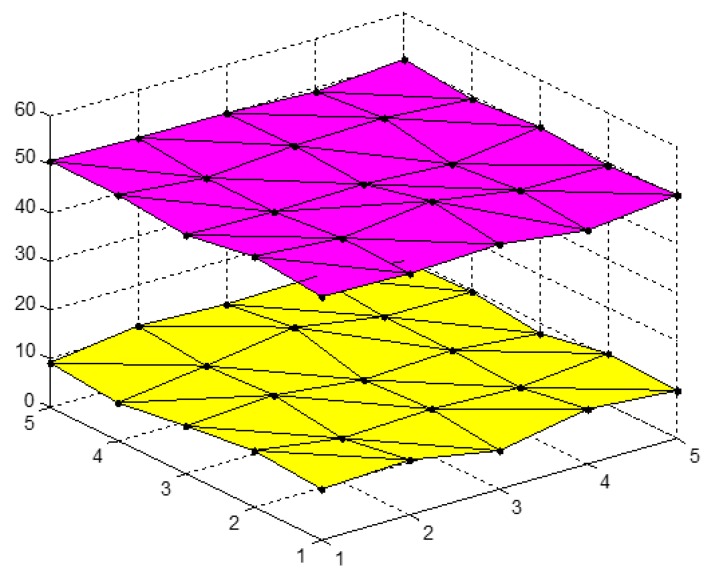
EGAR model panel data curved surfaces. The pink plane is generated by lining the panel data pots of research object and the yellow plane is generated by lining the panel data pots of another research object with one style.

**Figure 2:**
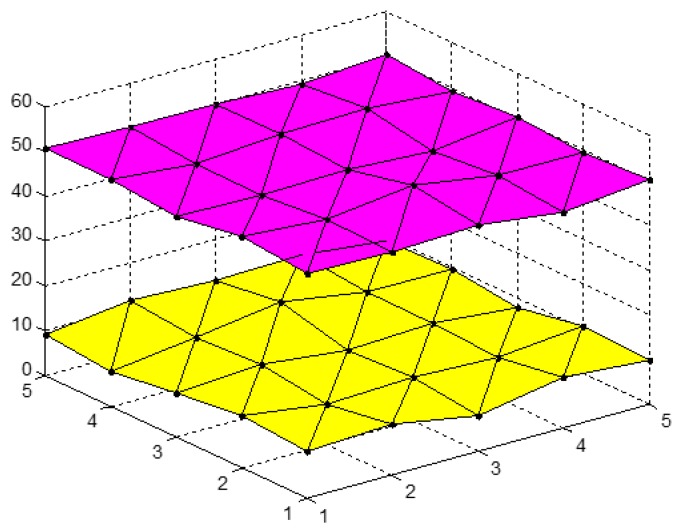
EGAR model panel data curved surfaces using different connections. The pink plane is generated by linking the panel data points of research object and the yellow plane is generated by linking the panel data points of another research object with another style.

Panel data are quite complex, containing both cross-sectional and time-series data that are characterized in both space and time dimensions. Panel data can intuitively be shown as disperse points in three-dimensional space. As shown in Figure 1 and Figure 2, the extended grey absolute relational (EGAR) model describes panel data as a series of curved surfaces in three-dimensional space. Based on the surface similarities, a relational degree can be defined.

First, assume that $X=\{X_{1}(s,t),\cdots ,X_{i}(s,t),\cdots ,X_{m}(s,t)|s=1,2,\cdots ,N;t=1,2,\cdots ,n\}$ is an index matrix sequence, and $X=\text{\{}A_{i}x_{i}+B_{i}y_{i}+C_{i}|i=1,2,\cdots ,m,x_{i}\in \left[s,s+1\right]$ $y_{i}\in \left[t,t+1\right],s=1,2,\cdots ,N-1;t=1,2,\cdots ,n-1\text{\}}$ are corresponding surface clusters [81]. Three adjacent elements in the index behaviour matrix, $X_{i}(s,t)$, form a triangle, and multiple triangle surfaces form a single index behaviour matrix. Thus, the panel data, X, can be expressed as a series of curved surfaces as shown previously in Figure 1 and Figure 2.

Next, the zero point image of the two indexes, $X_{i}(s,t)=(x_{i}(s,1),$ $x_{i}(s,2),\cdots ,x_{i}(s,n))$ and $X_{j}(s,t)=(x_{j}(s,1),x_{j}(s,2),\cdots ,x_{j}(s,n))$ for s∈[1,N], is defined as $X_{i}^{0}(s,t)=(x_{i}^{0}(s,1),$ $x_{i}^{0}(s,2),\cdots ,x_{i}^{0}(s,n))$ and $X_{j}^{0}(s,t)=(x_{j}^{0}(s,1),x_{j}^{0}(s,2),$ $\cdots ,x_{j}^{0}(s,n))$ [81]. If we let

$$s_{i}=\int_{1}^{N}\int_{1}^{n}X_{i}^{0}dxdy,$$

$$s_{j}=\int_{1}^{N}\int_{1}^{n}X_{j}^{0}dxdy,$$

and

$$s_{i}-s_{j}=\int_{1}^{N}\int_{1}^{n}(X_{i}^{0}-X_{j}^{0})dxdy,$$

the following equation can be used to determine the EGAR degree between two panel data:

(1) $$\varepsilon_{ij}=\frac{1+|s_{i}|+|s_{j}|}{1+|s_{i}|+|s_{j}|+|s_{i}-s_{j}|},$$

where $\left|s_{i}\right|$, $\left|s_{j}\right|$ and $\left|s_{i}-s_{j}\right|$ reflect the volumes between two zero point image surfaces and coordinate planes and the curved volume of two surfaces. The defined volumes in Figure 1 and Figure 2 are different, suggesting that the grey correlation degrees are not equal for two types of connecting methods and that non-unique results may occur.

To prove this supposition, consider a closed area *D*, where {s≤x≤s+1,t≤x≤t+1} for s=1,2,⋯,N−1 and t=1,2,⋯,n−1. Also, set

$$A_{1}=x_{i}^{0}(s,t),A_{2}=x_{i}^{0}(s+1,t),A_{3}=x_{i}^{0}(s,t+1),A_{4}=x_{i}^{0}(s+1,t+1)$$

$$B_{1}=x_{j}^{0}(s,t),B_{2}=x_{j}^{0}(s+1,t),B_{3}=x_{j}^{0}(s,t+1),B_{4}=x_{j}^{0}(s+1,t+1)$$

Considering the results presented in Figure 1, the volumes between the zero point images and the coordinate plane for the index matrix, $X_{i}$ and $X_{j}$, are as follows,

(2) $$|s_{i}|=|\sum_{s=1}^{N-1}\sum_{t=1}^{n-1}\frac{1}{3}(A_{2}+A_{3})+\sum_{s=1}^{N-1}\sum_{t=1}^{n-1}\frac{1}{6}(A_{1}+A_{4})|,$$

(3) $$|s_{j}|=|\sum_{s=1}^{N-1}\sum_{t=1}^{n-1}\frac{1}{3}(B_{2}+B_{3})+\sum_{s=1}^{N-1}\sum_{t=1}^{n-1}\frac{1}{6}(B_{1}+B_{4})|,$$

and the volume of the two curved surfaces can be determined as

(4) $$|s_{i}-s_{j}|=|\sum_{s=1}^{N-1}\sum_{t=1}^{n-1}\frac{1}{3}[(A_{2}+A_{3})-(B_{2}+B_{3})]+\sum_{s=1}^{N-1}\sum_{t=1}^{n-1}\frac{1}{6}[(A_{1}+A_{4})-(B_{1}+B_{4})]|.$$

Equation (1) can then be used to calculate $\varepsilon_{ij}$ as follows:

(5) $$\begin{array}{ll} \varepsilon_{ij}= & [6+|\sum_{s=1}^{N-1}\sum_{t=1}^{n-1}\left(2A_{2}+2A_{3}+A_{1}+A_{4}\right)|+|\sum_{s=1}^{N-1}\sum_{t=1}^{n-1}\left(2B_{2}+2B_{3}+B_{1}+B_{4}\right)|]\backslash \\ & \left\{6+|\sum_{s=1}^{N-1}\sum_{t=1}^{n-1}\left(2A_{2}+2A_{3}+A_{1}+A_{4}\right)|+|\sum_{s=1}^{N-1}\sum_{t=1}^{n-1}\left(2B_{2}+2B_{3}+B_{1}+B_{4}\right)\right|\\ & +|\sum_{s=1}^{N-1}\sum_{t=1}^{n-1}\left[2(A_{2}+A_{3}-B_{2}-B_{3}\right)+(A_{1}+A_{4}-B_{1}-B_{4})]|\} \end{array}.$$

Considering the results presented in Figure 2, the volumes between the zero point images and the coordinate plane for the index matrix, $X_{i}$ and $X_{j}$, are as follows,

(6) $$|s_{i}\prime |=|\sum_{s=1}^{N-1}\sum_{t=1}^{n-1}\frac{1}{6}(A_{2}+A_{3})+\sum_{s=1}^{N-1}\sum_{t=1}^{n-1}\frac{1}{3}(A_{1}+A_{4})|,$$

(7) $$|s_{j}\prime |=|\sum_{s=1}^{N-1}\sum_{t=1}^{n-1}\frac{1}{6}(B_{2}+B_{3})+\sum_{s=1}^{N-1}\sum_{t=1}^{n-1}\frac{1}{3}(B_{1}+B_{4})|,$$

and the volume of the two curved surfaces can be determined as

(8) $$|s_{i}\prime -s_{j}\prime |=|\sum_{s=1}^{N-1}\sum_{t=1}^{n-1}\frac{1}{6}[(A_{2}+A_{3})-(B_{2}+B_{3})]+\sum_{s=1}^{N-1}\sum_{t=1}^{n-1}\frac{1}{3}[(A_{1}+A_{4})-(B_{1}+B_{4})]|.$$

Equation (1) can then be used to calculate $\varepsilon_{ij}\prime$ as follows:

(9) $$\begin{array}{ll} \varepsilon_{ij}\prime = & [6+|\sum_{s=1}^{N-1}\sum_{t=1}^{n-1}\left(A_{2}+A_{3}+2A_{1}+2A_{4}\right)|+|\sum_{s=1}^{N-1}\sum_{t=1}^{n-1}\left(B_{2}+B_{3}+2B_{1}+2B_{4}\right)|]\backslash \\ & \left\{6+|\sum_{s=1}^{N-1}\sum_{t=1}^{n-1}\left(A_{2}+A_{3}+2A_{1}+2A_{4}\right)|+|\sum_{s=1}^{N-1}\sum_{t=1}^{n-1}\left(B_{2}+B_{3}+2B_{1}+2B_{4}\right)\right|\\ & +|\sum_{s=1}^{N-1}\sum_{t=1}^{n-1}\left[(A_{2}+A_{3}-B_{2}-B_{3}\right)+2(A_{1}+A_{4}-B_{1}-B_{4})]|\} \end{array}.$$

According to Equations (5) and (9), $\varepsilon_{ij}=\varepsilon_{ij}\prime$ if and only if $A_{1}=A_{2}=A_{3}=A_{4}=0$ and $B_{1}=B_{2}=B_{3}=B_{4}=0$ or comparably $x_{i}^{0}(s+1,t)=x_{i}^{0}(s,t+1)=x_{i}^{0}(s,t)=x_{i}^{0}(s+1,t+1)=0$ and $x_{j}^{0}(s+1,t)=$ $x_{j}^{0}(s,t+1)=x_{j}^{0}(s,t)=x_{j}^{0}(s+1,t+1)=0$. If these conditions are not met, non-unique EGAR results may occur.

The EGAR relational degree is measured as the volume difference between two curved surfaces; a lower volume difference suggests a higher correlation. Different connections affect the volume differences (i.e., different connections define different surfaces) and subsequently affect correlations between the same panel data. In addition, different sequences of investigation objects make relational results non-unique. To resolve this problem, we proposed an alternate grey relational method based on panel data.

## 3. Proposed DPGRA Model

### 3.1. DPGRA Model Principles

**Figure 3:**
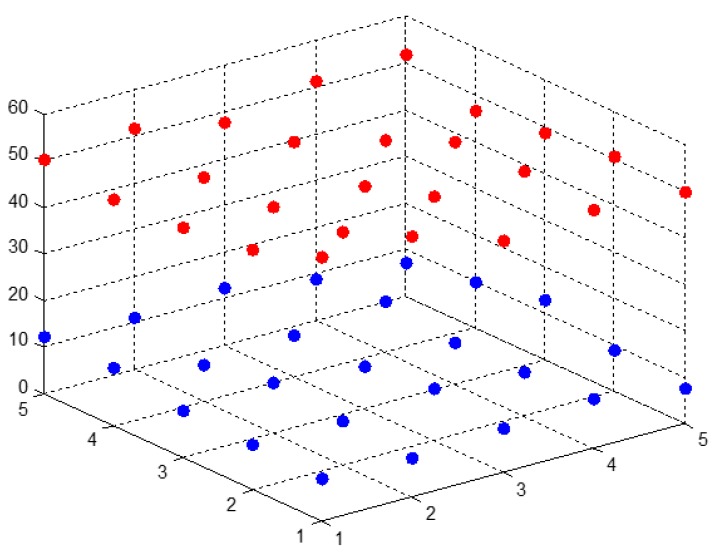
The scatter plot of panel data. The red dots are the panel data points of research object and the blue dots are the panel data points of another research object(these pots don’t link).

To support development of the proposed DPGRA model, a dispersion based on panel data was defined that not only characterized the dispersion of deviation from mean surface but also revealed the level of fluctuation in the samples. For sparsely distributed data, data volatility near the mean surface is higher, leading to a higher sum of squared differences between each data point and the mean and a higher dispersion. Conversely, a concentrated data distribution results in a lower sum of squared differences and a lower dispersion. Thus, a higher dispersion suggests higher data volatility and vice versa. Based on these dispersion characteristics, a grey relational degree based on panel data was developed that fully utilized information from the raw data. The scatter plot of panel data is shown in Figure 3.

#### 3.1.1. Dimensionless Processing

Before analyzing the grey relationship, data groups were transformed into dimensionless data. Beginning with the similarity measure of a data sequence, we used the following average transformation:

(10) $$X_{i}Z=\{x_{i}(1)z,x_{i}(2)z,\cdots ,x_{i}(n)z\},$$

where $x_{i}(k)z=\frac{x_{i}(k)}{{\bar{X}}_{i}}$, ${\bar{X}}_{i}=\frac{1}{n}\sum_{k=1}^{n}x_{i}(k)$, k=1,2,⋯,n, Z is the average operator and $X_{i}Z$ is the average change in $X_{i}$ when we set $X_{i}^{0}=\left\{x_{i}^{0}(1),x_{i}^{0}(2),\cdots x_{i}^{0}(n)\right\}=X_{i}Z$.

#### 3.1.2. Dispersion of Panel Data

Assuming that the values for indicators i(i=1,2,⋯,m) and j(j=1,2,⋯,m) for sample s(s=1,2,⋯,N) at time t(t=1,2,⋯,n) are $x_{i}(s,t)$ and $x_{j}(s,t)$, respectively, the behaviour matrix for index i is then

$$X_{i}(s,t)=[\begin{array}{cccc} x_{i}(1,1) & x_{i}(1,2) & \cdots & x_{i}(1,n)\\ x_{i}(2,1) & x_{i}(2,2) & \cdots & x_{i}(2,n)\\ \vdots & \vdots & \ddots & \vdots \\ x_{i}(N,1) & x_{i}(N,2) & \cdots & x_{i}(N,n) \end{array}].$$

the behaviour matrix for index j was similarly derived.

Averages were processed to determine $X_{i}^{0}(s,t)$ and $X_{j}^{0}(s,t)$. With corresponding distances of $l_{ij}(s,t)=|x_{i}^{0}(s,t)-x_{j}^{0}(s,t)|,$ for s=1,2,⋯,N and t=1,2,⋯,n, the resultant average distance is

$${\bar{l}}_{ij}=\frac{1}{N\times n}\sum_{s=1}^{N}\sum_{t=1}^{n}l_{ij}(s,t).$$

We then obtained the dispersion of panel data, *D*_ij_, as follows:

(11) $$D_{ij}=\frac{1}{N\times n}\sum_{s=1}^{N}\sum_{t=1}^{n}(l_{ij}(s,t)-{\bar{l}}_{ij}{)}^{2}.$$

#### 3.1.3. Panel Data Correlation

Again assuming that the values for indicators i(i=1,2,⋯,m) and j(j=1,2,⋯,m) for sample s(s=1,2,⋯,N) at time t(t=1,2,⋯,n) are $x_{i}(s,t)$ and $x_{j}(s,t)$, respectively, the results of Equation (11) were used to determine the grey relational degree based on dispersion between the $x_{i}(s,t)$ and $x_{j}(s,t)$ panels (DPGRA) as follows:

(12) $$\varepsilon_{ij}=\frac{\bar{D}}{\bar{D}+D_{ij}},$$

where $\bar{D}=\frac{1}{m}\sum D_{ij}$ is the mean of the dispersion. Based on this relationship, higher dispersions (indicated by higher $D_{ij}$ values) lead to smaller correlations among panel data $X_{i}(s,t)$ and $X_{j}(s,t)$.

### 3.2. DPGRA Model Properties

The proposed DPGRA model offers several advantageous properties, including normativity, symmetry, uniqueness and independence (i.e., $\varepsilon_{ij}$ is unique for two panel data matrices and is not influenced by other matrices) and comparability. To confirm these model properties, the following arguments are presented.

First, the required conditions for normativity, $0<\varepsilon_{ij}\leq 1$, $\varepsilon_{ij}=1⇐X_{i}^{0}(s,t)=X_{j}^{0}(s,t)$, are clearly established because $D_{ij}\in [0,+\infty )$. Second, the required conditions for symmetry, $\varepsilon_{ij}=\frac{\bar{D}}{\bar{D}+D_{ij}}=\varepsilon_{ji}=\frac{\bar{D}}{\bar{D}+D_{ji}}$, are met by the fundamental definition of the DPGRA degree. Third, uniqueness and independence are ensured by limiting the number of panel matrices considered in the determination of the dispersion. Each $\varepsilon_{ij}$ is determined from only two panel data matrices and is unaffected by other matrices in the system. This same DPGRA model principle confirms the requirements for comparability.

### 3.3. DPGRA Model Procedures

**Figure 4:**
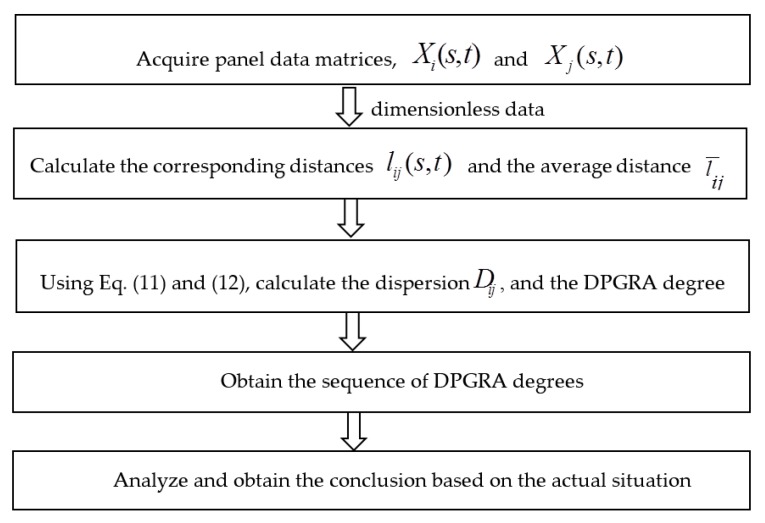
The steps diagram of DPGRA model.

The stepwise process for applying the proposed DPGRA model is as follows (Figure 4):

Acquire panel data matrices, $X_{i}(s,t)$ and $X_{j}(s,t)$, and obtain dimensionless data using Equation (10).

(1) Calculate the corresponding distances, $l_{ij}(s,t)$, for $X_{i}(s,t)$ and $X_{j}(s,t)$ as well as the average distance, ${\bar{l}}_{ij}$.
(2) Using Equations (11) and (12), calculate the dispersion, $D_{ij}$, and the DPGRA degree, $\varepsilon_{ij}$, respectively, and obtain the sequence of DPGRA degrees.
(3) Based on the disperse relational sequence, draw conclusions regarding quantitative relationships among investigation objects or indicators that complement direct analysis results.

## 4. Empirical Analysis Results

Most massive coastal disasters in the world are caused by storm surge. Countries bordering the northwest Pacific Ocean are particularly susceptible to storm surge disasters. Specifically, China experiences the most frequent and severe storm surges throughout the year and along the full extent of its coastline. In the 21st century, global climate change and growth in marine-related economic developments leads to an increased frequency of storm surges and concurrently ever-increasing economic losses, which adversely affect China coastal areas as well as the broader country. Thus, continued research regarding storm-tide disaster losses and response measures to reduce these losses is of both domestic and international significance.

### 4.1. Storm-Tide Disaster Loss Indexes

Storm-tide disasters that occur in the most populous and prosperous regions generally cause serious damage such as destroyed dykes, flooded oil fields, collapsed homes, farmland losses and damaged breeding. These outcomes seriously affect people’s lives and the economic prosperity of relevant industries. To best quantify these impacts, disaggregate economic losses should be considered.

In this study, we related direct economic losses to mariculture, coastal engineering, death toll, ship and collapsed home losses as well as storm surge frequency. This disaggregation provides the theoretical basis for reducing storm-tide disaster losses. We collected panel data for each of these variables from 2011 to 2015 (Tables S1–S7) in the five main coastal provinces of Jiangsu, Zhejiang, Fujian, Guangdong and Guangxi and subsequently modelled these data using the proposed DPGRA model.

As a first step in the analysis, we do the average treatment for each panel data variable (direct economic, mariculture, coastal engineering, death toll, ship and collapsed home losses as well as storm surge frequency) and transformed the data into dimensionless values. Next, using Equations (11) and (12), the dispersion, $D_{ij}$, and the DPGRA degree, $\varepsilon_{ij}$, were determined. Table 1 summarizes the dispersion results; individual calculations for the DPGRA degree are presented below.

With an estimated average dispersion of $\bar{D}=\frac{1}{6}\sum_{j=1}^{6}D_{0j}=3.6747$, estimated DPGRA degrees for each of the respective variables are as follows:

$$\varepsilon_{01}=\frac{\bar{D}}{\bar{D}+D_{01}}=0.7164,\;\varepsilon_{02}=\frac{\bar{D}}{\bar{D}+D_{02}}=0.7131,\;\varepsilon_{03}=\frac{\bar{D}}{\bar{D}+D_{03}}=0.2152,$$

$$\varepsilon_{04}=\frac{\bar{D}}{\bar{D}+D_{04}}=0.7608,\;\varepsilon_{05}=\frac{\bar{D}}{\bar{D}+D_{05}}=0.5145,\;\varepsilon_{06}=\frac{\bar{D}}{\bar{D}+D_{06}}=0.7705.$$

The proposed DPGRA model is insensitive to the relational data size and instead considers only the relative strength of the correlation degree (i.e., ranking order). The estimated DPGRA degree between storm-tide disaster direct economic losses and storm surge frequency was highest at 0.7705. The estimated DPGRA degrees between direct economic losses and other loss types were as follows, in rank descending order: ship (0.7608), mariculture (0.7164), coastal engineering (0.7131), collapsed home (0.5145) and death toll (0.2152). These estimates correspond to the estimated dispersion for panel data; higher dispersions result in lower correlations and vice versa.

#### 4.1.1. Comparison of Proposed DPGRA Model Results and True Data

A review of the raw or true data suggests that storm-tide disaster direct economic losses and storm surge frequencies exhibited similar variations, which subsequently suggests high correlation between these variables. Note that data from the Jiangsu Province from 2011 to 2015 did not show any variation and if this were not considered for further review. Based on a review of raw panel data from the remaining four provinces, direct economic losses and ship losses also exhibited similar variations, ranking this relationship second with respect to relational degree. Similarities in variations between direct economic losses and mariculture, coastal engineering and collapsed home losses were less apparent, ranking these relationships third, fourth and fifth with respect to relational degree. Conversely, death toll losses have 5 people in Guangdong in 2015, in the other five years, death toll losses of the main coastal city are 0, the overall fluctuation is very small; but direct economic losses of the five major coastal cities vary greatly from 2011 to 2015. Intuitively, the similarities between the direct economic losses and death toll losses are very tiny, that is, they have the lowest relational degree. Hence, this relationship was ranked sixth with respect to relational degree.

These collective observations from the raw or true data were consistent with and demonstrated the manifestation of true data in the proposed DPGRA model results. The estimated DPGRA degree between direct economic losses and storm surge frequency was also highest. Storm surge frequency reflects the storm-tide strength; hence, a stronger storm surge results in higher direct economic losses. Again consistent with true data observations, the estimated DPGRA degrees between direct economic losses and ship, mariculture and coastal engineering losses were each lower. Each is commonly tied to the sea: ships sail on the sea, mariculture development depends on the sea and coastal engineering structures are built near the sea. During a storm surge, offshore winds become stronger, waves become more powerful and sea levels rise, these result in heavy ship, mariculture and coastal engineering losses. Hence, their correlation with storm-tide disaster losses is relatively high. The estimated DPGRA degree between direct economic losses and collapsed home losses were lower, ranking fifth of six variables in both the true data and model estimates. Collapsed home losses are less tied to the sea; the extent of losses may be affected by the storm-tide intensity and a home distance from the sea. Finally, the estimated DPGRA degree between direct economic losses and death toll losses was also the lowest. Everyone has the own strong sense of protection, in the face of storm surge, the first consideration is their own security problems, and then again to consider their own property and other issues. Compared with other losses, death toll losses are smallest, so from the subjective view, the relational degree for ‘Death toll losses’ on economic losses due to storm-tide disaster in China is also lowest. The consistency of these results with the true data observations suggests that the proposed DPGRA model is valid and feasible.

#### 4.1.2. Comparison of the Proposed DPGRA Model and Conventional GRA Models

To further verify the proposed DPGRA model’s validity and feasibility, we compared its estimation results to the results from two conventional GRA models: (1) a Euclidean distance relational model (EDGRA) [81] and (2) a grey grid relational model (GGRA) [41]. Table 2 lists the estimated relational degrees from the proposed DPGRA and conventional GRA models. Table 3 lists the relative rankings of correlation between storm-tide disaster direct economic losses and mariculture, coastal engineering, death toll, ship and collapsed home losses as well as storm surge frequency.

The EDGRA model results indicated that the highest relational degree was between direct economic losses and collapsed home losses. The estimated EDGRA degrees between direct economic losses and other loss types were as follows, in rank descending order: death toll, mariculture, coastal engineering and ship losses and storm surge frequency. Estimates for collapsed home, mariculture, coastal engineering and ship losses were similar to estimates from the DPGRA model. Conversely, estimates for death toll losses and storm surge frequency were substantially different (opposite) from the DPGRA model. Different foundational theories may explain the differences in the two model results: the EDGRA model considers proximity but does not consider overall degree change and the DPGRA model considers proximity as well as overall volatility. For example, the death toll losses exhibited high proximity with direct economic losses but a high volatility. These combined characteristics likely affected the results reflected in DPGRA degrees.

Comparatively, results from the GGRA model showed that the relational degrees between direct economic losses and ship, coastal engineering, mariculture and collapsed home losses were highest. The lowest relational degree was between direct economic losses and death toll losses; this finding is roughly consistent with both the DPGRA model results and the true data. The relational degree between direct economic losses and storm surge frequency was ranked fifth in the GGRA model (and sixth in the EDGRA model). Comparatively, this relationship was ranked first in the DPGRA model, suggesting the highest relational degree. These differences in model results are likely attributable to the use of different measuring methods: the GGRA model is based on similar perspectives whereas DPGRA model considers comprehensive angles. Storm surge frequency ranks first in terms of the overall aspect but last in terms of similarity, leading to substantially divergent relative rankings in the GGRA and DPGRA models.

To summarise, the different relational models compared here yielded disparate relational results. However, the relational degrees between direct economic losses and ship, mariculture, coastal engineering and collapsed home losses were consistently relatively higher. These comparative results further confirm that the proposed DPGRA model is valid and feasible. What’s more, the traditional panel data relation is transformed into two-dimensional data relation, then make the average and obtain the final relational coefficient, but the average is too subjective, this doesn’t reflect the overall characteristics of panel data. As for DPGRA model, it can better solve this shortcoming. So DPGRA model is more reliable and feasible.

### 4.2. Storm-Tide Disaster Loss Objects

Storm surge events are largely unpredictable but have obvious regional characteristics. Using the proposed DPGRA model, we investigated these regional characteristics by comparing relationships among China’s five main coastal provinces of Jiangsu, Zhejiang, Fujian, Guangdong and Guangxi using seven indicators (direct economic, mariculture, coastal engineering, death toll, ship and collapsed home losses and storm surge frequency).

Using Equations (11) and (12), we calculated the dispersion, $D_{ij}$, and the DPGRA degree, $\varepsilon_{ij}$, for each of the five provinces. Table 4 and Table 5 summarize these results, respectively.

**Figure 5:**
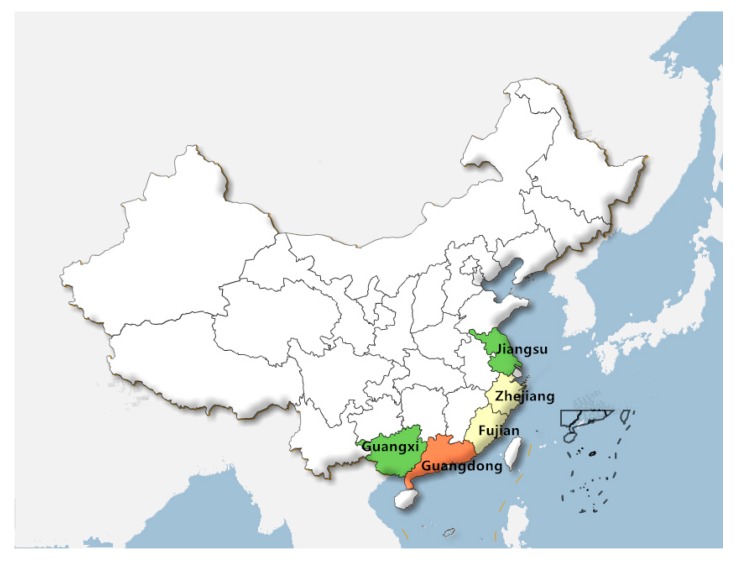
The regional characteristics of China coast main five provinces about storm surge. Different colors show that the relational degree of every loss and economic loss is different, the same color show the relational degree is similar.

The DPGRA model results in Table 5 reflect a diagonal matrix, in which the diagonal values are each equal to one. Thus, we focused on correlation among the five provinces rather than correlation with the five provinces themselves. The estimated DPGRA degrees were highest between Fujian and Zhejiang (0.6992) and to a lesser extent Guangxi and Jiangsu (0.5377). The estimated DPGRA degrees among other provinces were relatively low. Both Fujian and Zhejiang are near the East China Sea with coastline lengths of 3023.6 and 2253.7 km, respectively. At these locations, storm surge frequency and intensity are analogical. Similarly, Guangxi is near the South China Sea with a coastline length of 1478.2 km and Jiangsu is near China’s Yellow Sea with a coastline length of 1039.7 km, resulting in comparable storm surge effects. Although Guangdong is also near the South and East China Seas, has a coastline length of 4314.1 km and experiences serious storm surge effects, this province did not exhibit high correlations with any other province. The regional characteristics of China coast main five provinces about storm surge are shown in Figure 5. Therefore, this comparative analysis not only further confirmed the validity and feasibility of the proposed PRGRA model but also proved its ability to highlight regional differences in need of further investigation.

### 4.3. Summary of Empirical Analysis Results

Based on the proposed DPGRA model, the empirical analysis results showed that the relational degree between storm-tide disaster direct economic losses and storm surge frequency was highest. The estimated DPGRA degrees between direct economic losses and ship, mariculture, coastal engineering and collapsed home losses were each lower (in rank order) but still relatively high. The estimated DPGRA degree between direct economic losses and death toll losses was lowest. Caused by natural factors, storm surge events are inevitable. As such, minimizing direct economic losses by reducing storm surge frequency is not a feasible response strategy. Instead, storm-tide disaster response measures intended to reduce economic losses in China should focus on the protection of ship, mariculture, coastal engineering and building structures. We recommend the following three measures:

(1) Support advance protection of ship and mariculture structures (before the storm surge event) by improving storm-tide disaster forecasting and prediction systems. At present, the primary storm surge forecasting method in China is numerical prediction; the precision of these predictions is affected by research methods and available hydrological and meteorological data. Implementation of additional tidal stations and enhancements to marine satellite and fixed-point measurement systems would provide additional data and improve the numerical precision of forecasts. Alerting those responsible for ship and mariculture structures based on these early forecasts is essential for mitigating and preventing damage.
(2) Improve the standards of coastal engineering construction. Coastal areas are often fast developing and densely populated; in the event of a storm surge, losses are significant. Thus, engineering design parameters should be more stringent in these key areas, particularly with respect to protective structures such as seawalls, dykes and levees for flood control.
(3) Similarly, improve the standards of residential construction in areas susceptible to frequent storm-tide disasters. Homes are intended to shelter people from storms; when homes collapse because of a storm-tide disaster, both economic losses and personal hardship result. Thus, more stringent design requirements related to construction materials and structure height in areas susceptible to storm surge events may help to minimise these losses.

## 5. Conclusions

Conventional GRA models based on panel data can produce non-unique results for the same data sets. To overcome this issue, we proposed a GRA model based on the dispersion of panel data. Specifically, the DPGRA model was developed based on the dispersion of panel data. The proposed model’s properties, including uniqueness, symmetry and normalization, were characterized. Results indicated that the proposed DPGRA model not only improved upon conventional GRA model deficiencies but also revealed the potential volatility of investigation objects and the overall similarities in panel data. To demonstrate its applicability, the proposed DPGRA model was used to the relational analysis about storm-tide disaster losses in China coastal areas. The proposed model’s feasibility and validity were illustrated using different panel data indexes and objects.

To date, China has made significant advances in disaster research and implementation in response to floods, earthquakes and other natural disasters. Despite these advances, deficiencies in response to marine disasters persist. Using our proposed DPGRA model, we were able to relate storm-tide disaster direct economic losses to various disaggregate types of losses (ship, mariculture, coastal engineering, and collapsed home and death toll losses) as well as storm surge frequency. Results indicated that ship, mariculture, coastal engineering and collapsed home losses were most closely correlated with direct economic losses in the event of a storm-tide disaster. Death toll losses were not highly correlated. Storm surge frequency was most highly correlated with direct economic losses, but response strategies were unable to affect the occurrence of storm surge events.

Thus, to minimize the direct economic losses caused by storm surge events, the protection of ship, mariculture, coastal engineering and building structures should be a priority. Specifically, efforts to better forecast threats and alert those responsible for ship and mariculture structures in advance of a storm surge event should be improved. In addition, more stringent design, construction and reinforcement requirements for coastal engineering (e.g., seawalls, dykes, levees) and residential structures in areas susceptible to storm-tide disasters should be enacted. These focused response efforts have the potential to significantly reduce the direct economic losses caused by storm surge events. The results of this study provide a theoretical basis for implementing these response strategies and significantly contribute to the state of knowledge regarding storm-tide disaster impacts.

Although the DPGRA model proposed in this study proved valid and feasible, we will continue to improve this model by considering the dynamic characteristics of panel data relationships. We will also apply the proposed DPGRA model to different areas and fields in order to confirm the practical significance of the model.

## Figures and Tables

**Table 1:** Estimated dispersion between direct economic losses and various loss types and storm surge frequencies.

Results	Loss Type					Storm Surge Frequency
	Mariculture	Coastal Engineering	Death toll	Ship	Collapsed Home	
	*D*_01_	*D*_02_	*D*_03_	*D*_04_	*D*_05_	*D*_06_
**Dispersion**	1.4547	1.4778	13.3995	1.1552	3.4671	1.0942

**Table 2:** Estimated relational degrees from the proposed DPGRA model and two conventional GRA models.

Loss Type	DPGRA Degree	EDGRA Degree	GGRA Degree
Mariculture losses	0.7164	0.8322	0.8164
Coastal engineering losses	0.7131	0.8247	0.8229
Death toll losses	0.2152	0.8651	0.6979
Ship losses	0.7608	0.8054	0.8360
Collapsed home losses	0.5145	0.8693	0.7979
Storm surge frequency	0.7705	0.7434	0.7590

**Table 3:** Relative rankings of correlation between direct economic losses and various loss types and storm surge frequencies for the proposed DPGRA model and two conventional GRA models.

Loss Type	DPGRA Degree	EDGRA Degree	GGRA Degree
Mariculture losses	3	3	3
Coastal engineering losses	4	4	2
Death toll losses	6	2	6
Ship losses	2	5	1
Collapsed home losses	5	1	4
Storm surge frequency	1	6	5

**Table 4:** Estimated dispersion among China’s five main coastal provinces.

Results	Jiangsu	Zhejiang	Fujian	Guangdong	Guangxi
Jiangsu	0				
Zhejiang	18.0646	0			
Fujian	15.157	3.4956	0		
Guangdong	17.1004	9.4189	10.3014	0	
Guangxi	6.9854	14.7555	14.6588	11.9532	0

**Table 5:** Estimated DPGRA degree among China’s five main coastal provinces.

Results	Jiangsu	Zhejiang	Fujian	Guangdong	Guangxi
Jiangsu	1				
Zhejiang	0.3102	1			
Fujian	0.3490	0.6992	1		
Guangdong	0.3221	0.4632	0.4410	1	
Guangxi	0.5377	0.3551	0.3566	0.4047	1

## Supplementary Materials

The following are available online at www.mdpi.com/1660-4601/14/11/1330/s1, Table S1: Storm tide direct economic losses of China five coastal provinces, 2011–2015 (million yuan), Table S2: Mariculture losses of China five coastal provinces, 2011–2015 (thousands of hectares), Table S3: Coastal engineering losses of China five coastal provinces, 2011–2015 (kilometers), Table S4: The death toll of China five coastal provinces, 2011–2015 (person), Table S5: Ship losses of China five coastal provinces, 2011–2015 (ship), Table S6: Collapsed houses number of China five coastal provinces, 2011–2015 (room), Table S7: Storm surge frequency of China five coastal provinces, 2011–2015 (number).
