# Supplementary material for: Research on Storm-Tide Disaster Losses in China Using a New Grey Relational Analysis Model with the Dispersion of Panel Data
Source: Int J Environ Res Public Health. 2017 Nov 1;14(11):1330. doi: 10.3390/ijerph14111330 (PMC5707969; doi:10.3390/ijerph14111330)
Supplement: Supplementary file 1 [file ijerph-14-01330-s001.pdf]

# A Novel Grey Relational Analysis Model with the Dispersion of Panel Data and its Application to Chinese Storm-Tide Disaster Losses

**Kedong Yin, Ya Zhang, Xuemei Li**

**Table S1.** Storm tide direct economic losses of China's five coastal provinces, 2011-2015 (million yuan).

| Province | Years | | | | |
|---|---|---|---|---|---|
| | **2011** | **2012** | **2013** | **2014** | **2015** |
| Jiangsu | 0.61 | 6.15 | 0.17 | 0.47 | 0.58 |
| Zhejiang | 1.92 | 42.57 | 28.17 | 4.33 | 11.2 |
| Fujian | 5.32 | 2.64 | 45.06 | 4.29 | 30.79 |
| Guangdong | 12.63 | 17.47 | 74.2 | 60.41 | 28.76 |
| Guangxi | 1.15 | 5.33 | 4.9 | 28.3 | 0.47 |

**Table S2.** Mariculture losses of China's five coastal provinces, 2011-2015 (thousands of hectares)

| Province | Years | | | | |
|---|---|---|---|---|---|
| | **2011** | **2012** | **2013** | **2014** | **2015** |
| Jiangsu | 7.8 | 42.28 | 2.26 | 12.75 | 0.02 |
| Zhejiang | 0 | 48.47 | 37.81 | 9.55 | 27.44 |
| Fujian | 1.62 | 5.78 | 54.85 | 14.19 | 27.54 |
| Guangdong | 17.4 | 5.88 | 37.38 | 45.37 | 24.43 |
| Guangxi | 4.44 | 103.81 | 0.62 | 8.83 | 1.59 |

**Table S3.** Coastal engineering losses of China's five coastal provinces, 2011-2015 (kilometers)

| Province | Years | | | | |
|---|---|---|---|---|---|
| | **2011** | **2012** | **2013** | **2014** | **2015** |
| Jiangsu | 9.5 | 20.1 | 6.37 | 23.78 | 0 |
| Zhejiang | 13.75 | 259.39 | 29.55 | 6.74 | 41.28 |
| Fujian | 0.05 | 3.87 | 125.02 | 13.67 | 88.71 |
| Guangdong | 2.15 | 6.15 | 100.38 | 20.37 | 29.01 |
| Guangxi | 22.75 | 21.07 | 7.64 | 75.97 | 4.47 |

**Table S4.** The death toll of China's five coastal provinces, 2011-2015 (person)

| Province | Years | | | | |
|---|---|---|---|---|---|
| | **2011** | **2012** | **2013** | **2014** | **2015** |
| Jiangsu | 0 | 0 | 0 | 0 | 0 |
| Zhejiang | 0 | 0 | 0 | 0 | 0 |
| Fujian | 0 | 0 | 0 | 0 | 0 |
| Guangdong | 0 | 9 | 0 | 0 | 5 |
| Guangxi | 0 | 0 | 0 | 0 | 0 |

**Table S5.** Ship losses of China's five coastal provinces, 2011-2015 (ship)

| **Province** | **Years** | | | | |
|---|---|---|---|---|---|
| | **2011** | **2012** | **2013** | **2014** | **2015** |
| Jiangsu | 0 | 0 | 0 | 0 | 0 |
| Zhejiang | 148 | 915 | 2124 | 202 | 639 |
| Fujian | 21 | 167 | 6066 | 413 | 4265 |
| Guangdong | 303 | 506 | 5382 | 1213 | 2325 |
| Guangxi | 0 | 1 | 32 | 501 | 0 |

**Table S6.** Number of collapsed houses of China's five coastal provinces, 2011-2015 (room)

| **Province** | **Years** | | | | |
|---|---|---|---|---|---|
| | **2011** | **2012** | **2013** | **2014** | **2015** |
| Jiangsu | 0 | 233 | 0 | 0 | 127 |
| Zhejiang | 0 | 0 | 175 | 60 | 116 |
| Fujian | 200 | 6 | 1101 | 53 | 3858 |
| Guangdong | 600 | 1391 | 4001 | 11717 | 78 |
| Guangxi | 0 | 718 | 620 | 0 | 72 |

**Table S7.** Storm surge frequency of China's five coastal provinces, 2011-2015 (number)

| **Province** | **Years** | | | | |
|---|---|---|---|---|---|
| | **2011** | **2012** | **2013** | **2014** | **2015** |
| Jiangsu | 1 | 2 | 2 | 3 | 1 |
| Zhejiang | 1 | 3 | 3 | 1 | 3 |
| Fujian | 1 | 2 | 5 | 3 | 3 |
| Guangdong | 1 | 4 | 3 | 3 | 2 |
| Guangxi | 1 | 3 | 3 | 2 | 2 |

**Data sources:** China marine disasters communique
